# Prevalence of adolescent deliveries and its complications in Cameroon: a systematic review and meta-analysis

**DOI:** 10.1186/s13690-020-00406-1

**Published:** 2020-05-05

**Authors:** Tsi Njim, Bayee Swiri Tanyitiku, Carlson Sama Babila

**Affiliations:** 1 Health and Human Development (2HD) Research Group, Douala, Littoral region Cameroon; 2 Higher Institute of Commerce and Management, University of Bamenda, Bamenda, North west region Cameroon; 3 Clinical Research Education, Networking and Consultancy, Douala, Cameroon

**Keywords:** Adolescent deliveries, Adolescent pregnancies, Maternal complications, Foetal complications, Cameroon

## Abstract

**Background:**

Adolescent deliveries (10–19 years) carry a high risk of adverse outcomes due to the biological and physiological immaturity of these mothers. They pose a significant health burden in Cameroon, as it is reported that a high proportion of women attending delivery services are teenagers. We therefore sought to systematically assess the prevalence of adolescent deliveries in the country and its maternal and neonatal outcomes.

**Methods:**

This was a systematic review of literature and a meta-analysis. We searched MEDLINE, CINAHL and Global Health online databases for all studies that reported the proportion of adolescent women who presented for delivery in health facilities in Cameroon. All observational studies published up to 10th July 2019, were included.

**Results:**

A total of 47 articles were identified by the search. After removal of duplicates and screening of the titles and abstracts, 11 eligible studies were retained with ten articles meeting the inclusion criteria. These ten studies finally retained reported on nine different cohorts with a total of 99,653 women. The pooled prevalence of adolescent deliveries from the nine cohorts in Cameroon was 14.4% (95% CI: 10.7–18.6%), the prevalence for early adolescent deliveries was 2.8% (95% CI: 0.4–7.2%), meanwhile that for late adolescent deliveries was 12.5% (95% CI: 6.7–19.8%). The prevalence of adolescent deliveries in urban areas – 13.1% (95% CI: 7.8–19.6%) was similar to that in semi-urban areas– 14.1% (95% CI: 6.7–23.5%). Adolescents were more likely than adults (> 19 years) to have low birth weight babies (OR: 1.8; 95% CI: 1.6, 2.1); babies born with asphyxia (OR: 1.7; 95% CI: 1.3, 2.1); babies born before term (OR: 1.5; 95% CI: 1.1, 1.9) and babies who die in the neonatal period (OR: 2.1; 95% CI: 1.2, 3.8).

**Conclusion:**

The prevalence of adolescent deliveries in Cameroon is high. Implementation of adolescent-friendly policies is necessary to reduce the proportion of adolescents who become pregnant in Cameroon.

## Background

Adolescent deliveries carry a high risk of adverse outcomes to both the mother and the baby largely due to a biological and physiological immaturity; particularly pelvic and perineal immaturity in adolescents [1–3]. Some of the adverse outcomes of adolescent deliveries to the mother include: a higher rate of caesarean sections, perineal trauma and maternal death; while neonates born to adolescent mothers are likely to suffer from asphyxia, low birth weight and neonatal death [1, 4–10]. The mortality associated with adolescent deliveries and the long-term morbidity due to its adverse outcomes like low birth weight (which has a known relationship with neurodevelopmental complications) [11], makes adolescent births a public health issue.

In Cameroon, the health burden of adolescent pregnancies and deliveries is significant with a high proportion of women attending delivery services being teenagers [12]. This prevalence of adolescent deliveries varies greatly in different regions of the country; with studies showing a prevalence of 9.3–9.9% [6, 9, 13] in several urban settings and as high as 26.5% recorded in the north of the country and some rural areas where early marriages are engraved in the cultural values [1, 10].

A previous systematic review and meta-analysis of adolescent pregnancies in Africa gave an estimate of adolescent pregnancies in Cameroon at 16.31% [14]. However, this review did not include all the studies that have been carried out in the country and did not provide the complications of adolescent deliveries and pregnancies. Providing an estimate of the prevalence of adolescent deliveries in Cameroon (and the prevalence in the various regions of the country) and its various adverse events will help policymakers to prioritise resources and set out strategies to reduce the associated burdens of the condition. This could also help clinicians anticipate the various adverse events during adolescent deliveries which could lead to early management and success. We therefore seek to conduct this review to systematically assess the prevalence and adverse events of adolescent deliveries in the various regions of Cameroon.

### Definitions

Adolescence deliveries or teenage deliveries: deliveries occurring between the maternal ages of 10–19 years and at a gestational age of greater than 28 weeks.

Early adolescent deliveries: deliveries occurring at maternal ages of 10–16 years while late adolescent deliveries occur at maternal ages of 17–19 years.

Adult women: generally defined as women aged > 19 years.

Preterm deliveries: deliveries occurring from 28 to 36 weeks of gestation; term deliveries from 37 to 42 weeks of gestation; while post-term deliveries occur above 42 weeks of gestation.

Neonatal asphyxia: defined using the Apgar score. Babies with Apgar scores < 7 are considered to have asphyxia.

## Methods

This review was carried out following a predesigned review protocol that has been e-published in the PROSPERO registration database: registration number - CRD42019142020. We report our findings according to Preferred Reporting Items for Systematic Reviews and Meta-Analysis (PRISMA) guidelines (Additional file 1).

### Study design and eligibility criteria

This was a systematic review of published studies which included all studies (cohort studies, cross-sectional studies, case-control studies) that reported on the proportion of women who deliver during adolescence in Cameroon (Table 1). Studies that reported on birth outcomes in these women were also included in the review.

**Table 1:** PICOS strategy for inclusion criteria of studies into review

PICOS strategy	Inclusion criteria	
**P-population**	Pregnant women who present for deliveries across hospitals in Cameroon	
**I-intervention/Exposure**	Pregnant women who present for delivery during their adolescent years (13–19 years)	
**C-comparison**	Pregnant adult women presenting for delivery	
**O-outcome(s)**	Maternal complications	caesarean deliveries, perineal tears, operative vaginal deliveries, post-partum haemorrhage and episiotomies
	Foetal complications	low birth weight, asphyxia, preterm, neonatal period, post-term, stillbirth, high birth weight
**S-study design**	All observational studies	

### Exclusion criteria

We excluded:

- Same studies published in different journals with the same or a different title. Studies or manuscripts with several publications of findings over time had the most recent of the findings were chosen;
- Letters to the editor, reviews, editorials and commentaries;
- Conference abstracts of unpublished studies which had a measure of retention of patients in care or attrition;

### Setting

Cameroon is a country in Central Africa made up of ten geopolitical regions – the Far north, north, Adamawa, South west, North west, Littoral, Centre, West, East and South regions (Additional file 2). It has an estimated population of 26,223,783 inhabitants with most of them being subsistence farmers – and an unemployment rate of 4.4% [15, 16]. The gross domestic product per capita is 1446.70 Unites States dollars per capita [15].

### Information sources

We searched the following online databases: Medline, CINAHL and Global Health using predefined search strategies for relevant abstracts of articles (Additional file 3). The search strategies used in the review were developed using Boolean operators. The main search terms included “Pregnancy In Adolescence” [Mesh terms], combined with Cameroon [Mesh terms].

Articles returned by the search were saved to EndNote version × 8 software which was used to remove duplicates. The titles and abstracts of the articles obtained after exclusion of duplicates were assessed for eligibility using the inclusion and exclusion criteria by two independent reviewers (TN and BST). Disagreements were settled by consensus. Full text of the abstracts and titles deemed eligible for the review were retrieved and assessed in detail independently by the reviewers. The reference lists of these articles were searched to obtain similar articles.

### Data management

After assessment of the full texts, data from the articles that were included in the review were extracted by the two independent reviewers; using a tool that was developed and pretested by TN prior to the commencement of the database search on Microsoft Excel 2010 software.

### Data items and extraction

Using the established data extraction sheets, the two primary reviewers extracted the following information from each article included in the final review for interpretation: Last name of first author; date of publication; region in which study was conducted; study design; setting (urban versus rural); type of health facility (primary, secondary or tertiary); age range of participants; prevalence of adolescent deliveries; type of adolescent delivery (early versus late); maternal sociodemographic, clinical and obstetric characteristics (marital status, gravidity, parity, educational status, occupational status and number of antenatal care visits attended); and various maternal (preterm delivery; post-partum haemorrhage, perineal tears, caesarean delivery, post-term delivery, operative delivery and episiotomies) and neonatal complications (asphyxia, stillbirth, neonatal death, low birth weight and high birth weight).

### Assessment of methodological quality and risk of bias

The two reviewers independently assessed the methodological quality and risk of bias for each study. Any discrepancies were settled by consensus between the two reviewers. Assessment was done using the Quality Assessment Tool for Observational Studies of the National Health Institute/National Heart, Lung, and Blood Institute made up of 14 questions as detailed in Additional File 4. The studies were divided into three categories. Studies were said to be of “good quality” if > 70% of the applicable criteria were attained in the quality assessment tool; “fair quality” if > 50% of the applicable criteria were attained in the quality assessment tool and “poor quality” if ≤50% of the applicable criteria were attained in the quality assessment tool.

### Data synthesis and analysis

The prevalence of adolescent deliveries from each study was described and a meta-analysis was used to obtain an overall pooled prevalence of adolescent deliveries in the different regions and in Cameroon as a whole. Due to the possibility of heterogeneity among the studies due to the variability of the different regions, designs and populations; both a random and fixed effects meta-analysis models were reported, but the random models were used for interpretation. The chi-squared test for heterogeneity and the I^2^ statistic were used to assess the degree of heterogeneity among the various studies; where 25, 50, and 75% were used to represent low, moderate and high heterogeneity respectively. Subgroup analyses were also done to obtain pooled effects for different groups: region, setting and late versus early adolescent deliveries.

Meta-analyses were also performed to obtain overall estimates (odds ratios) for the sociodemographic, clinical and obstetric characteristics of adolescent mothers and the various maternal and neonatal adverse events. Pooled estimates were only obtained if more than two studies reported an estimate.

After the meta-analysis, a conceptual framework which included the birth outcomes of adolescent mothers in Cameroon was produced to provide a theoretical interpretation to the relationship between adolescent mothers and adverse birth outcomes.

## Results

**Fig. 1:**
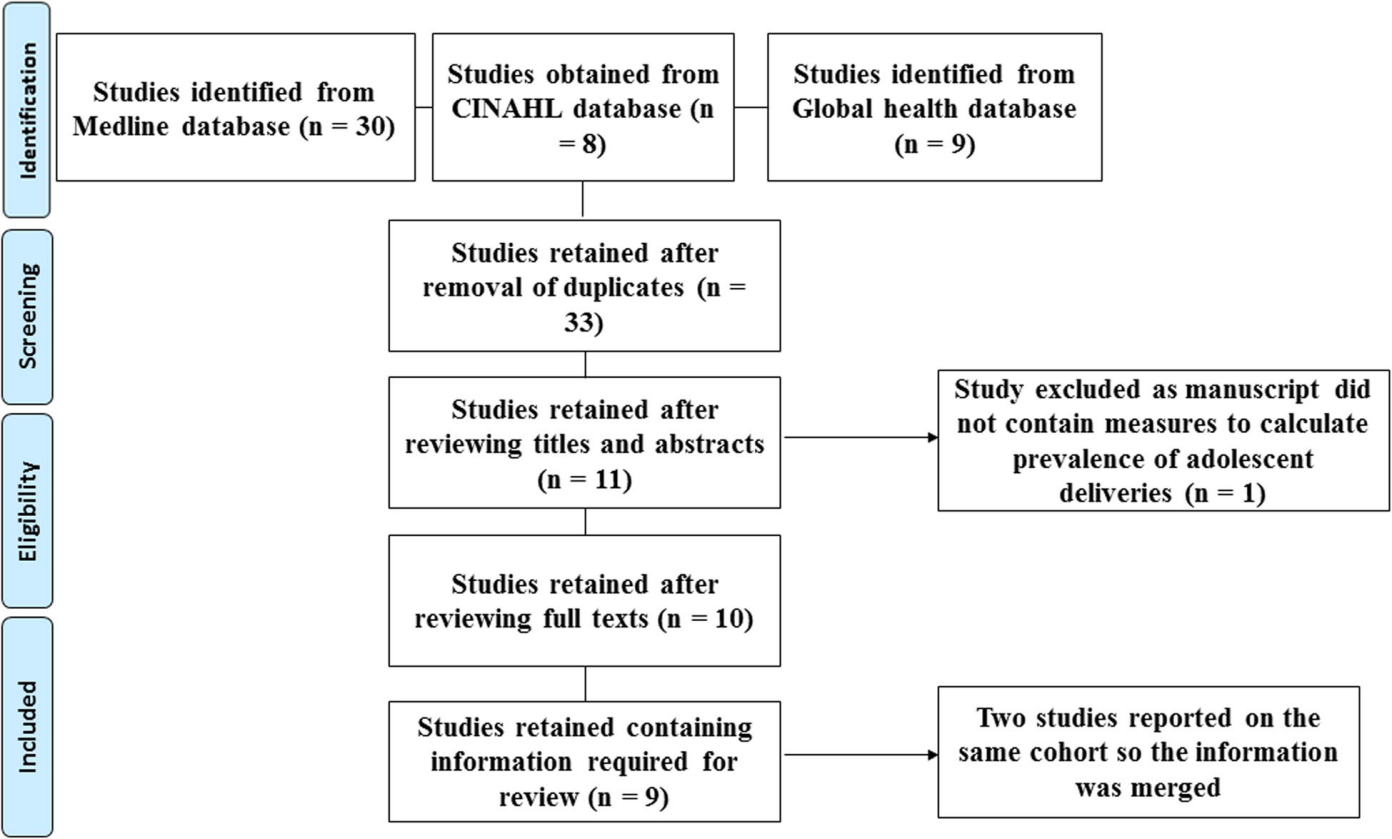
PRISMA flow diagram showing inclusion and exclusion of studies in the review

A total of 47 articles were initially identified by the search (Fig. 1). After removal of duplicates 33 studies remained. Screening of the titles and abstracts eliminated 22 irrelevant articles. The full texts of 11 eligible studies were scrutinized with ten articles meeting the inclusion criteria. These ten studies finally retained reported on nine different cohorts of women with a total of 99,653 women. Two of the studies reported on the same cohort of women – one reporting on birth outcomes in women and the other on birth outcomes in the children [6, 13]. Two studies provided estimates for the prevalence of adolescent deliveries in the North west region [1, 2], two studies provided estimates for the South west region [3, 17], two studies for the Centre region [6, 7, 13], one for the Littoral region [9], one for the Far north region [10] and one study provided an estimate from all ten regions in the country [12]. All the studies used to assess the prevalence of adolescent deliveries were cross-sectional studies but Egbe et al used a case-control design in the second phase of their study to determine the maternal and foetal outcomes of adolescent deliveries [17].

Using the Quality Assessment Tools for observational studies of the National Health Institute/National Heart, Lung, and Blood Institute; seven of the articles describing six cohorts of women were of “good quality” [1–3, 6, 7, 9, 13] while the other three were designated to be of “fair quality” [10, 12, 17] (Appendix 4).

The characteristics of the studies included in the review are summarised in Table 2.

**Table 2:** Characteristics of studies included in systematic review

Author name and year	Sample size	Number of adolescents	Years of recruitment	Type of study	Region	Age profile	Type of health facility	Setting
Agbor, 2017 [1]	1803	368	2009–2016	Retrospective register analysis	North west	14–49	Primary hospital	Rural
Egbe, 2015 [17]	6546	874	2010–2013	Retrospective register analysis & case-control	South west	Not clear	Secondary hospital	Semiurban
Tamambang, 2018 [9]	8056	662	2010–2015	Retrospective register analysis	Littoral	26.6 ± 6.4	Tertiary hospitals	Urban
Njim, 2017 [2]	886	77	2015–2016	Retrospective register analysis	North west	26.6 ± 5.3	Secondary hospital	Semiurban
Njim, 2016 [3]	4941	491	2007–2012	Retrospective register analysis	South west	26.4 ± 5.5	Secondary hospital	Semiurban
Kongnyuy, 2008 [7]	1100	268	2004–2005	Cross-sectional study	Centre	20–29	Tertiary hospitals	Urban
Tebeu, 2010 [12]	57,787	8222	2003–2005	Retrospective register analysis	All ten regions	Not clear	Various	Various
Tebeu, 2006 [10]	12,537	3328	1995–2004	Retrospective register analysis	Far north	Not clear	Secondary hospital	Semiurban
Fouelifack, 2014 [6, 13]	5997	560	2008–2010	Retrospective register analysis	Centre	27.34 ± 6.0	Tertiary hospitals	Urban

Not clear: Mean age not provided or age range did not have upper limit for the adult women

### Prevalence of adolescent deliveries

**Fig. 2:**
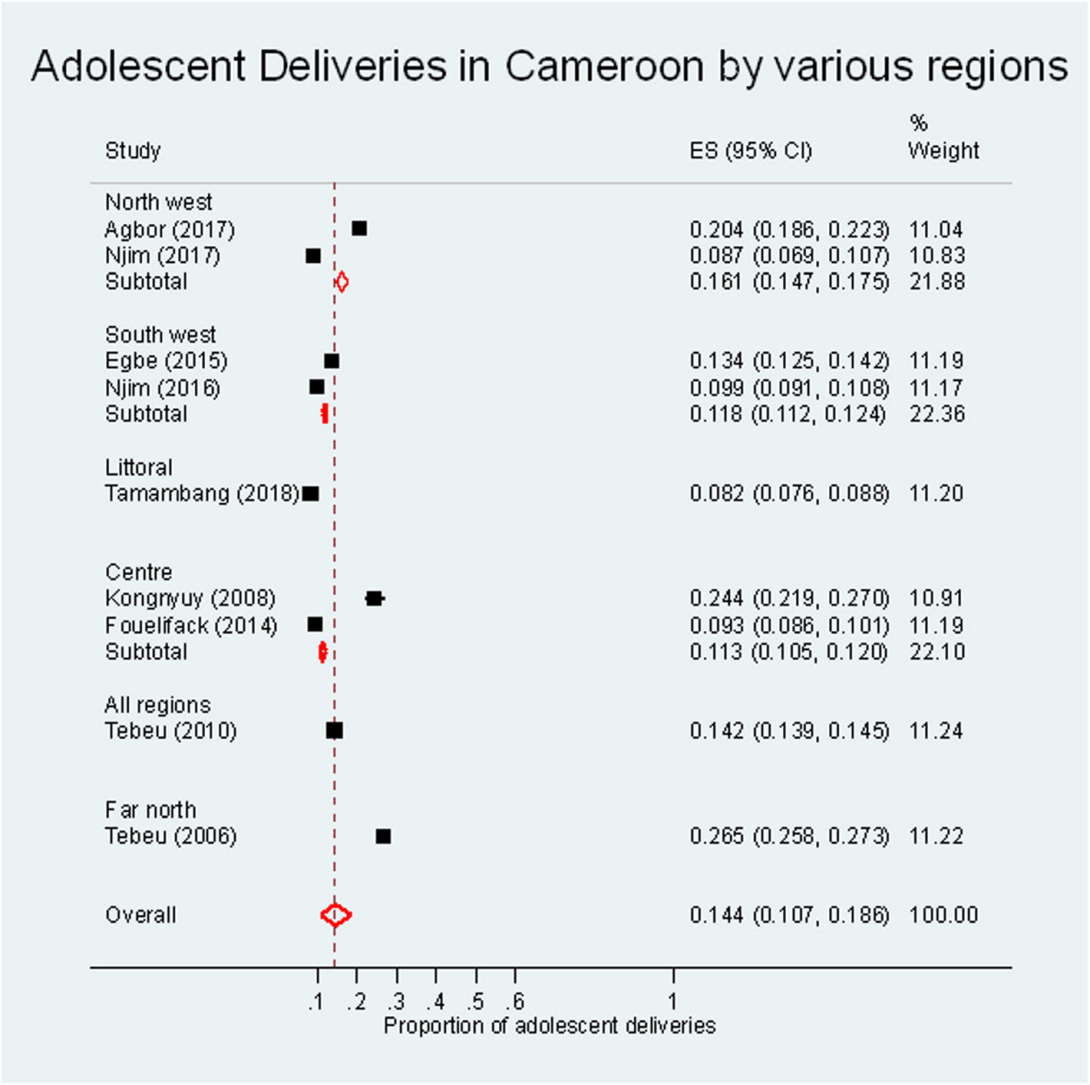
Meta-analysis of the prevalence of adolescent deliveries in Cameroon

The pooled prevalence of adolescent deliveries from the nine cohorts in Cameroon was 14.4% (95% CI: 10.7–18.6%, I^2^ = 99.6%, χ^2^ = 1861.9, *p* < 0.001). Figure 2 shows the estimates for the various regions. Four studies reported on the prevalence of early and late adolescent deliveries [1, 3, 9, 10]. The overall pooled estimate for early adolescent deliveries was 2.8% (95% CI: 0.4–7.2%, I^2^ = 99.6%, χ^2^ = 812.7, *p* < 0.001), meanwhile that for late adolescent deliveries was 12.5% (95% CI: 6.7–19.8%, I^2^ = 99.6%, χ^2^ = 747.5, *p* < 0.001) (Additional File 5). The prevalence of adolescent deliveries in urban areas [6, 7, 9, 13] – 13.1% (95% CI: 7.8–19.6%) was similar to that in semi-urban areas [2, 3, 10, 17] – 14.1% (95% CI: 6.7–23.5%) (I^2^ = 99.6%, χ^2^ = 47.4, *p* < 0.001) (Additional File 5). Similarly, there was no difference in the prevalence of adolescent deliveries in secondary hospitals [2, 3, 10, 17] when compared with tertiary hospitals [6, 7, 9, 13] [14.1% (95% CI: 6.7, 23.5%) vs 13.1% (95% CI: 7.8, 19.6%); I^2^ = 99.6%, χ^2^ = 47.4, p < 0.001] (Additional File 5).

### Sociodemographic and obstetric factors of adolescent deliveries

After meta-analyses, adolescents were more likely to be primigravidas (OR: 12.5; 95% CI: 4.6, 33.5; I^2^ = 92.0%, χ^2^ = 39.9, *p* < 0.001) [1, 6, 9, 17]; while adolescents were just as likely to be single [1–3, 6, 7, 17], primiparous [1, 2, 6, 9] and unemployed [6, 7, 17] as adult women (Additional File 6).

Two studies described the level of education and its association with adolescent deliveries. Egbe et al [17] showed that adolescents in a semi-urban population were just as likely to have a primary level of education as adult women (OR: 1.8; 95% CI: 0.9, 3.6) and Kongnyuy et al [7] determined that in an urban population adolescents were more than four times more likely to have only a primary level of education when compared with adult women (OR: 4.5; 95% CI: 3.2, 6.2) (Additional File 6).

These two studies also described the association of number of antenatal care visits with adolescent deliveries; with Kongnyuy et al [7] showing no difference in the number of visits between adolescents and adult women and Egbe et al [17] showing that adolescents were two times more likely to have attended less than four visits when compared to adult women (OR: 2.1; 95% CI: 1.4, 3.1) (Additional File 6).

### Maternal complications of adolescent deliveries

After pooling estimates for maternal complications of adolescent deliveries, adolescents were just as likely to have caesarean deliveries (OR: 1.0; 95% CI: 0.7, 1.4; I^2^ = 55.0%, χ^2^ = 11.1, *p* = 0.05) [1–3, 7, 9, 13]; perineal tears (OR: 1.9; 95% CI: 0.7, 5.4; I^2^ = 68.0%, χ^2^ = 6.2, *p* = 0.05) [1, 7, 13] and operative vaginal deliveries (OR: 1.1; 95% CI: 0.1, 10.4; I^2^ = 87%, χ^2^ = 22.8, *p* < 0.01) [3, 7, 9, 13] when compared with adult women (Additional File 7).

Two studies assessed the relationship between adolescent deliveries and post-partum haemorrhage and both showed that adolescents were just as likely to have post-partum haemorrhage as adult women [1, 6] (Additional File 7). Two studies also assessed the proportion of episiotomies in women at delivery. Kongnyuy et al [7] showed that adolescents were more likely to have episiotomies at delivery (OR: 1.8; 95% CI: 1.2, 2.7) while Fouelifack et al [6] showed that adolescents were just as likely to have episiotomies at delivery as adult women (OR: 1.1; 95% CI: 0.8, 1.4) (Additional File 7).

### Foetal and neonatal complications of adolescent deliveries

Adolescents were more likely to have low birth weight babies (OR: 1.8; 95% CI: 1.6, 2.1; I^2^ = 0%, χ^2^ = 2.1, *p* = 0.8) [1–3, 7, 9, 17]; babies born with asphyxia (OR: 1.7; 95% CI: 1.3, 2.1; I^2^ = 27%, χ^2^ = 8.2, *p* = 0.22) [1–3, 7, 9, 13, 17]; babies born before term (OR: 1.5; 95% CI: 1.1, 1.9; I^2^ = 70.0%, χ^2^ = 19.8, *p* < 0.01) [1–3, 7, 9, 13, 17] and babies who die in the neonatal period (OR: 2.1; 95% CI: 1.2, 3.8; I^2^ = 0%, χ^2^ = 0.5, *p* = 0.8) [2, 7, 17] (Additional File 8).

Babies born to adolescent mothers were just as likely to be born in the post-term period [1, 3, 9, 13, 17] and as a stillbirth [1, 3, 7, 9, 13, 17] as babies born to adult mothers (Additional File 8). These babies were also less likely to have a high birth weight (OR: 0.4; 95% CI: 0.3, 0.6; I^2^ = 0%, χ^2^ = 2.1, *p* = 0.55) when compared with babies from adult mothers [1–3, 9] (Additional File 8).

### Conceptual framework

**Fig. 3:**
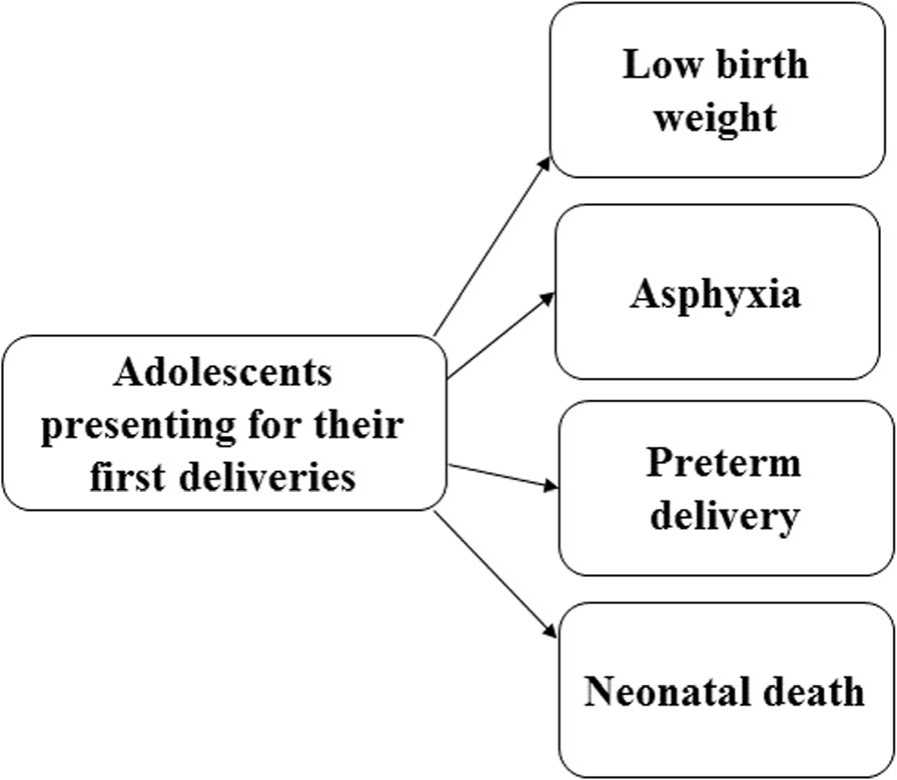
Conceptual framework of adolescent deliveries in Cameroon

The conceptual framework of adolescent deliveries in Cameroon is shown in Fig. 3.

## Discussion

In this review, we determined that the national prevalence of adolescent deliveries in Cameroon was 14.4%. The prevalence in the various regions ranged from 11.3% in the Centre region to 16.1% in the North west region. Adolescents who presented with their first deliveries were more likely to have low birth weight babies, babies born before term, with asphyxia and who died in the neonatal period.

The prevalence of adolescent deliveries in Cameroon is high. This is however similar to the prevalence in other West and Central African countries like Nigeria (15.9%), Ghana (12.2%) and Gabon (23.7%) [14]. In developed countries, the prevalence of adolescent deliveries is lower with countries like Sweden reporting a prevalence of 1.6% in 2003 and the Netherlands with a prevalence of 4% [18, 19]. Several authors have hypothesised that the high prevalence of adolescent deliveries in low and middle-income settings could be attributed to inadequate education on sexual and reproductive health amongst adolescents in schools, and the low awareness of appropriate contraceptive methods in these populations [3, 4, 20]. Also, adolescents in these settings are less likely to use reproductive health services [4]. In Cameroon, the present curriculum in schools and colleges lacks depth in sexual health education. This could therefore point to the need for more adolescent friendly sexual health programmes and education to raise awareness amongst teenagers and reduce the prevalence of deliveries amongst teenagers in Cameroon.

In this review, adolescents presenting for delivery were just as likely to be married as adult women. In most rural areas and some regions like the Far north of the country, adolescent marriages are common practice [1, 8, 10, 12]. This could explain why the proportions of married adolescents presenting for deliveries was similar to that of married adult women. As expected, adolescents in the studies were also presenting for the deliveries in their first pregnancy.

Adolescents were just as likely to have caesarean deliveries, perineal tears and operative vaginal deliveries as adult women. The association between caesarean deliveries and adolescent deliveries has been controversial with some authors stating that adolescents were more likely to have deliveries through caesarean sections [21] while other authors found contrary findings [5, 6, 13, 22]. Studies which found a positive association attributed this finding to biological and perineal immaturity in adolescents and the lack of proper antenatal care in this population [23]. However, some authors have shown that studies that have high finding of caesarean sections in adolescents was due to the healthcare providers and mothers considering that this mode of delivery is much safer [24]. The prevalence of caesarean sections in some regions in Cameroon could be as low as 6.1% in some regions [9]. This might show a tendency of caesarean sections being used as a last resort, hence, why no significant difference exists in the proportions of adolescents and adult women who have caesarean sections.

Adolescents were also more likely to have preterm deliveries and low birth weight infants when compared with adult women. The association between adolescent deliveries and low birth weight may not be a direct relationship. Spontaneous preterm deliveries have been shown to be associated with maternal undernutrition [22]. Such biological factors like low prenatal weight gain due to undernutrition coupled with sociodemographic factors like poverty could lead to spontaneous preterm deliveries hence low birth weight [22, 25, 26]. Babies who are born preterm with low birth weight are at a higher risk of having neonatal asphyxia and future neurocognitive developmental problems. In this review, we also found an association between adolescent births and neonatal asphyxia.

Survival in neonates is much lower when they are born with low birth weight, preterm delivery and asphyxia and the risk of morbidity is much higher in this group of infants [22, 27]. This is due to the higher probability of complications like respiratory distress, low birth weight and hypothermia in these neonates [27]. This could explain the association between adolescent deliveries and neonatal death shown in this review.

### Implications

Due to the high prevalence of adolescent deliveries, it is required that policies geared towards reducing adolescent pregnancies should be investigated and implemented. Presently, there are no specific policies targeted towards the reduction of adolescent pregnancies in Cameroon as detailed by Agbor et al [1]. Furthermore, as stated above, the current educational curriculum in secondary schools is deficient in sexual education and fails to address the sexual needs of adolescents. Adolescent-friendly educational programmes in these schools which goes into details about issues like contraception could help reduce the prevalence of adolescent pregnancies in the country.

### Limitations

This review attempted to provide a national and regional estimate for adolescent deliveries in Cameroon. However, there was no separate published data for five regions in the country. The study carried out by Tebeu et al in 2010 [12], collated data from all ten regions in the country. Even though the authors did not provide an independent estimate for adolescent deliveries in each region, the average estimate obtained from their study (14.2%) was similar to that obtained in this review (14.4%). There was a high degree of heterogeneity when pooling the results from all the studies as the populations that were studied were different. Cameroon is a multi-ethnic society with over 250 different ethnicities clustered into the ten different regions. These regions also have diverse cultural and environmental influences that make the subpopulations different.

## Conclusion

The prevalence of adolescent deliveries in Cameroon is high. There is a need to implement adolescent-friendly health and sexual education programmes by policymakers as this may reduce the proportion of adolescents who become pregnant in Cameroon. Also, clinicians should anticipate complications of adolescent deliveries (low birth weight, preterm delivery, neonatal asphyxia and neonatal death) to help draw up management plans and guidelines; which could decrease the morbidity and mortality associated with this condition.

## Supplementary information

**Additional file 1.** Preferred Reporting Items for Systematic Reviews and Meta-Analyses (PRISMA) Checklist. Checklist which shows the details of all aspects of a systematic review included in the review.

**Additional file 2.** Map of Cameroon. Map of Cameroon showing the ten geopolitical regions of the country – Far north, North, Adamawa, Centre, East, South, Littoral, South west, North west and west regions.

**Additional file 3.** Electronic searches. Electronic searches of online databases showing number of articles assessed.

**Additional file 4.** Methodological and risk of bias assessment. Methodological and risk of bias assessment using the Quality Assessment Tool for Observational Studies of the National Health Institute/National Heart, Lung, and Blood Institute.

**Additional file 5.** Meta-analysis showing prevalence of adolescent deliveries in Cameroon. Meta-analysis showing the prevalence of adolescent deliveries by early vs late adolescent deliveries, setting and type of health facility.

**Additional file 6.** Meta-analysis of risk factors of adolescent deliveries. Meta-analysis of the various risk factors of adolescent deliveries in Cameroon.

**Additional file 7.** Meta-analysis of maternal complications of adolescent deliveries. Meta-analysis of the various maternal complications of adolescent deliveries in Cameroon.

**Additional file 8.** Meta-analysis of the foetal/neonatal complications of adolescent deliveries. Meta-analysis of the various foetal/neonatal complications of adolescent deliveries in Cameroon.

## Data Availability

Not applicable.

## Abbreviation

OR: Odds ratio

## References

[1] Agbor VN, Mbanga CM, Njim T (2017). Adolescent deliveries in rural Cameroon: an 8-year trend, prevalence and adverse maternofoetal outcomes. Reprod Health.

[2] Njim T, Agbor VN. Adolescent deliveries in semi-urban Cameroon: prevalence and adverse neonatal outcomes. BMC Res Notes. 2017;10(227) (26 June 2017)-(26 June). 10.1186/s13104-017-2555-3 PMC5485502 28651611

[3] Njim T, Choukem S-P, Atashili J, Mbu R (2016). Adolescent deliveries in a secondary-level Care Hospital of Cameroon: a retrospective analysis of the prevalence, 6-year trend, and adverse outcomes. J Pediatr Adolesc Gynecol.

[4] Amoran OE (2012). A comparative analysis of predictors of teenage pregnancy and its prevention in a rural town in Western Nigeria. Int J Equity Health.

[5] Eriksen JK, Clapp M, Melamed A, Little S, Zera C. Cesarean delivery in adolescent pregnancies. Am J Gynecol. 2015. 10.1016/j.jpag.2016.01.123 26836505

[6] Fouelifack FY, Tameh TY, Mbong EN, Nana PN, Fouedjio JH, Fouogue JT, et al. Outcome of deliveries among adolescent girls at the Yaoundé central hospital. BMC Pregnancy Childbirth. 2014;14(102) (17 March 2014)-(17 March). 10.1186/1471-2393-14-102 PMC3995430 24636077

[7] Kongnyuy EJ, Nana PN, Fomulu N, Wiysonge SC, Kouam L, Doh AS (2008). Adverse perinatal outcomes of adolescent pregnancies in Cameroon. Matern Child Health J.

[8] Njim T, Agbor VN. Adolescent deliveries in rural Cameroon: comparison of delivery outcomes between primipara and multipara adolescents. BMC Res Notes. 2018;11(427) (3 July 2018)-(3 July). 10.1186/s13104-018-3550-z PMC6029040 29970162

[9] Tamambang RF, Njim T, Njie AE, Mbuagbaw L, Mafuta A, Tchana M (2018). Adolescent deliveries in urban Cameroon: a retrospective analysis of the prevalence, 6-year trend and adverse outcomes. BMC Res Notes.

[10] Tebeu PM, Tantchou J, Obama Abena MT, Mevoula Onala D, Leke RJI (2006). Delivery outcome of adolescents in far North Cameroon. Revue Medicale De Liege.

[11] Wadhwa PD, Buss C, Entringer S, Swanson JM (2009). Developmental origins of health and disease: brief history of the approach and current focus on epigenetic mechanisms. Semin Reprod Med.

[12] Tebeu PM, Kemfang JD, Sandjong DI, Kongnyuy E, Halle G, Doh AS. Geographic distribution of childbirth among adolescents in Cameroon from 2003 to 2005. Obstet Gynecol Int. 2010;2010. 10.1155/2010/805165 PMC2926687 20798773

[13] Fouelifack FY, Fouedjio JH, Fouogue JT, Fouelifa LD, Nguefack FD, Mbu ER (2015). Fetal outcome of deliveries among teenagers in Centre region of Cameroon. Br J Med Med Res.

[14] Kassa GM, Arowojolu AO, Odukogbe AA, Yalew AW (2018). Prevalence and determinants of adolescent pregnancy in Africa: a systematic review and meta-analysis. Reprod Health.

[15] The World Bank. The World Bank Data 2019. Available from: https://data.worldbank.org/country/cameroon. Accessed 7 Jan 2020.

[16] World Population Review. World Population Review - Cameroon 2019. Available from: http://worldpopulationreview.com/countries/cameroon-population/#popDensityMap. Accessed 7 Jan 2020.

[17] Omeichu A, Halle-Ekane GE, Tchente CN, Egbe E-N, Oury J-F, Egbe TO (2015). Prevalence and outcome of teenage hospital births at the Buea Health District, south west region, Cameroon. Reprod Health.

[18] Tyrberg RB, Blomberg M, Kjolhede P (2013). Deliveries among teenage women - with emphasis on incidence and mode of delivery: a Swedish national survey from 1973 to 2010. BMC Pregnancy Childbirth.

[19] World Health Organisation (2004). Adolescent pregnancy: issues in adolescent health and development.

[20] Amoran OE, Oluwole FA, Salami OF (2012). A comparative analysis of teenagers and older pregnant women in the utilization of prevention of mother to child transmission [PMTCT] services in, Western Nigeria. BMC Int Health Hum Rights.

[21] Ezegwui HU, Ikeako LC, Ogbuefi F (2012). Obstetric outcome of teenage pregnancies at a tertiary hospital in Enugu, Nigeria. Niger J Clin Pract.

[22] Marvin-Dowle K, Kilner K, Burley VJ, Soltani H (2018). Impact of adolescent age on maternal and neonatal outcomes in the born in Bradford cohort. BMJ Open.

[23] Mahavarkar SH, Madhu CK, Mule VD (2008). A comparative study of teenage pregnancy. J Obstet Gynaecol.

[24] da Gama SGN, Viellas EF, Corrêa Schilithz AO, Theme Filha MM, Lazaro de Carvalho M, Oliveira Gomes KR, et al. Factors associated with caesarean section among primiparous adolescents in Brazil, 2011-2012. Cad Saúde Pública [online]. 2014;30(Supplement 1). 10.1590/0102-311x00145513 25167171

[25] Roth J, Hendrickson J, Schilling M, Stowell DW (1998). The risk of teen mothers having low birth weight babies: implications of recent medical research for school health personnel. J Sch Health.

[26] Goldenberg RL, Culhane JF, Iams JD, Romero R (2008). Epidemiology and causes of preterm birth. Lancet..

[27] Njim T, Atashili J, Mbu R, Choukem SP (2015). Low birth weight in a sub-urban area of Cameroon: an analysis of the clinical cut-off, incidence, predictors and complications. BMC Pregnancy Childbirth..

