# Supplementary material for: Prevalence of adolescent deliveries and its complications in Cameroon: a systematic review and meta-analysis
Source: Arch Public Health. 2020 May 5;78:24. doi: 10.1186/s13690-020-00406-1 (PMC7199297; doi:10.1186/s13690-020-00406-1)
Supplement: Supplementary file 2 — Additional file 2. Map of Cameroon. Map of Cameroon showing the ten geopolitical regions of the country – Far north, North, Adamawa, Centre, East, South, Littoral, South west, North west and west regions. [file 13690_2020_406_MOESM2_ESM.pdf]

Map of Cameroon showing the ten geopolitical regions of the country – Far north, North, Adamawa, Centre, East, South, Littoral, South west, North west and west regions.

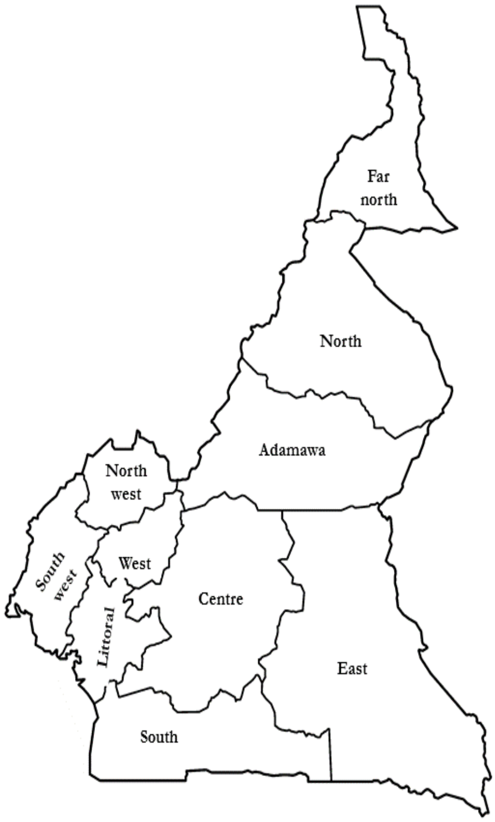

*Not to scale

Map adapted from Wikimedia: https://www.kisspng.com/png-regions-of-cameroon-map-wikimedia-commons-atlas-of-1460917/download-png.html
