# Supplementary material for: Prevalence of adolescent deliveries and its complications in Cameroon: a systematic review and meta-analysis
Source: Arch Public Health. 2020 May 5;78:24. doi: 10.1186/s13690-020-00406-1 (PMC7199297; doi:10.1186/s13690-020-00406-1)
Supplement: Supplementary file 3 — Additional file 3. Electronic searches. Electronic searches of online databases showing number of articles assessed. [file 13690_2020_406_MOESM3_ESM.pdf]

Information sources

Electronic sources

Online database: Medline was searched using the search strategy below from the date of creation of the database to 10/07/2019, for relevant abstracts of articles.

**Searches**	**Search combinations**	**Search terms**	**Number of hits**
S1		“MH Pregnancy In Adolescence+”	7,654
S2		"Pregnancy in adolescence" OR "adolescen* pregnan*" OR “teen* pregnan*” OR "adolescen* deliver*" OR "teen deliver*" OR "young maternal pregnan*" OR "young maternal deliver*" OR “youth pregnan*” OR “youth deliver*”	9,529
S3	S1 OR S2		9,529
S4		“MH Cameroon+”	5,061
S5		Cameroon	9,890
S6	S4 OR S5		9,890
S7	S3 AND S6		30

Online database: CINAHL was searched using the search strategy below from the date of creation of the database to 10/07/2019, for relevant abstracts of articles.

**Searches**	**Search combinations**	**Search terms**	**Number of hits**
S1		“MH Pregnancy In Adolescence+” OR (MH "Maternal Age 14 and Under")	5,089
S2		"Pregnancy in adolescence" OR "adolescen* pregnan*" OR “teen* pregnan*” OR "adolescen* deliver*" OR "teen deliver*" OR "young maternal pregnan*" OR "young maternal deliver*" OR “youth pregnan*” OR “youth deliver*”	5,789
S3	S1 OR S2		5,810
S4		“MH Cameroon+”	1,025
S5		Cameroon	1,273

S6	S4 OR S5		1,273
S7	S3 AND S6		8

Online database: Global health was searched using the search strategy below from the date of creation of the database to 10/07/2019, for relevant abstracts of articles.

**Searches**	**Search combinations**	**Search terms**	**Number of hits**
S1		"Pregnancy in adolescence" OR "adolescen* pregnan*" OR “teen* pregnan*” OR "adolescen* deliver*" OR "teen deliver*" OR "young maternal pregnan*" OR "young maternal deliver*" OR “youth pregnan*” OR “youth deliver*”	1,262
S2		Cameroon	8,689
S3	S1 AND S2		9
