# Supplementary material for: Prevalence of adolescent deliveries and its complications in Cameroon: a systematic review and meta-analysis
Source: Arch Public Health. 2020 May 5;78:24. doi: 10.1186/s13690-020-00406-1 (PMC7199297; doi:10.1186/s13690-020-00406-1)
Supplement: Supplementary file 4 — Additional file 4. Methodological and risk of bias assessment. Methodological and risk of bias assessment using the Quality Assessment Tool for Observational Studies of the National Health Institute/National Heart, Lung, and Blood Institute. [file 13690_2020_406_MOESM4_ESM.pdf]

Methodological and risk of bias assessment using the Quality Assessment Tool for Observational Studies of the National Health Institute/National Heart, Lung, and Blood Institute

	Criteria	Agbor, 2017	Egbe, 2015	Tamambang, 2018	Njim, 2017	Njim, 2016	Kongnyuy, 2008	Tebeu, 2010	Tebeu, 2006	Fouelifack, 2014 & 2015
1	Was the research question or objective in this paper clearly stated?	Yes	Yes	Yes	Yes	Yes	Yes	Yes	Yes	Yes
2	Was the study population clearly specified and defined?	Yes	Yes	Yes	Yes	Yes	Yes	Yes	Yes	Yes
3	Was the participation rate of eligible persons at least 50%?	Yes	Yes	Yes	Yes	Yes	Yes	Yes	Yes	Yes
4	Were all the subjects selected or recruited from the same or similar populations (including the same time period)? Were inclusion and exclusion criteria for being in the study prespecified and applied uniformly to all participants?	Yes	Yes	Yes	Yes	Yes	Yes	Yes	Yes	Yes
5	Was a sample size justification, power description, or variance and effect estimates provided?	No	No	No	No	No	No	No	No	No
6	For the analyses in this paper, were the exposure(s) of interest measured prior to the outcome(s) being measured?	Yes	Yes	Yes	Yes	Yes	Yes	Yes	Yes	Yes
7	Was the timeframe sufficient so that one could reasonably expect to see an association between exposure and outcome if it existed?	NA	NA	NA	NA	NA	NA	NA	NA	NA
8	For exposures that can vary in amount or level, did the study examine different levels of the exposure as related to the outcome (e.g., categories of exposure, or exposure measured as continuous variable)?	NA	NA	NA	NA	NA	NA	NA	NA	NA
9	Were the exposure measures (independent variables) clearly defined, valid, reliable, and implemented consistently across all study participants?	Yes	Yes	Yes	Yes	Yes	Yes	Yes	Yes	Yes
10	Was the exposure(s) assessed more than once over time?	No	No	No	No	No	No	No	No	No
11	Were the outcome measures (dependent variables) clearly defined, valid, reliable, and implemented consistently across all study participants?	Yes	No	Yes	Yes	Yes	Yes	No	No	Yes

12	Were the outcome assessors blinded to the exposure status of participants?	No	No	No	No	No	NC	NC	NC	NC
13	Was loss to follow-up after baseline 20% or less?	NA	NA	NA	NA	NA	NA	NA	NA	NA
14	Were key potential confounding variables measured and adjusted statistically for their impact on the relationship between exposure(s) and outcome(s)?	Yes	Yes	Yes	Yes	Yes	Yes	No	No	Yes
	Quality rating									
	Rater #1 TN	Good	Fair	Good	Good	Good	Good	Fair	Fair	Good
	Rater #2 BST	Good	Fair	Good	Good	Good	Good	Fair	Fair	Good

*Not clear
