# Supplementary material for: Prevalence of adolescent deliveries and its complications in Cameroon: a systematic review and meta-analysis
Source: Arch Public Health. 2020 May 5;78:24. doi: 10.1186/s13690-020-00406-1 (PMC7199297; doi:10.1186/s13690-020-00406-1)

Prevalence of early adolescent deliveries in Cameroon

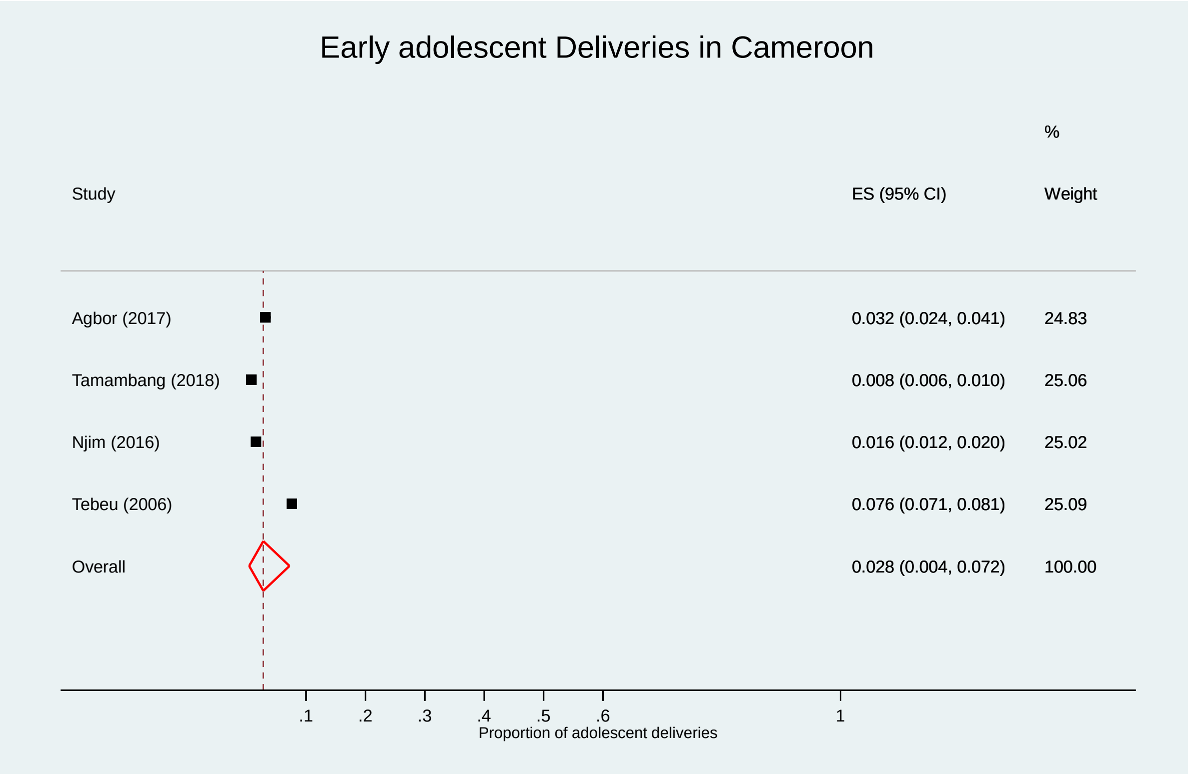

Prevalence of late adolescent deliveries in Cameroon

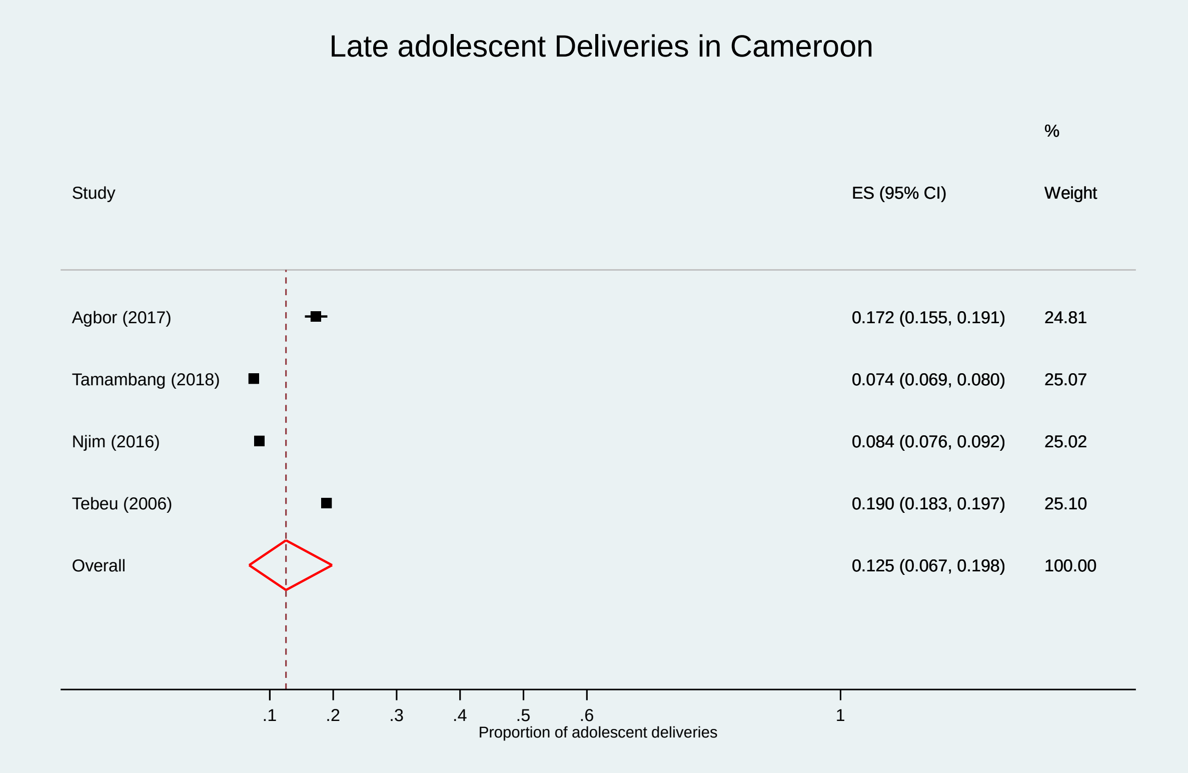

Prevalence of adolescent deliveries in Cameroon by setting (rural vs semi-urban vs urban)

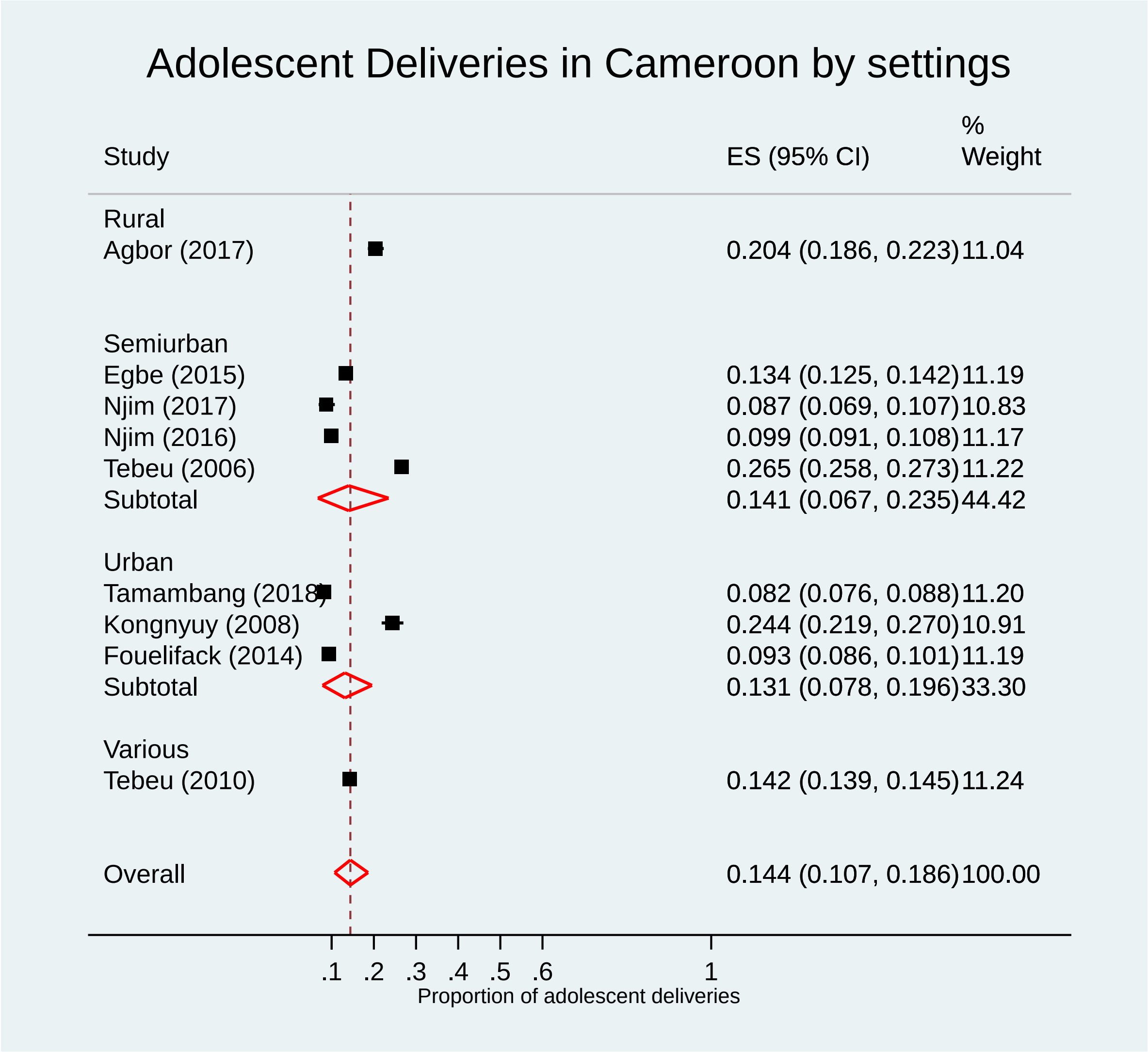

Prevalence of adolescent deliveries in Cameroon by type of health facility

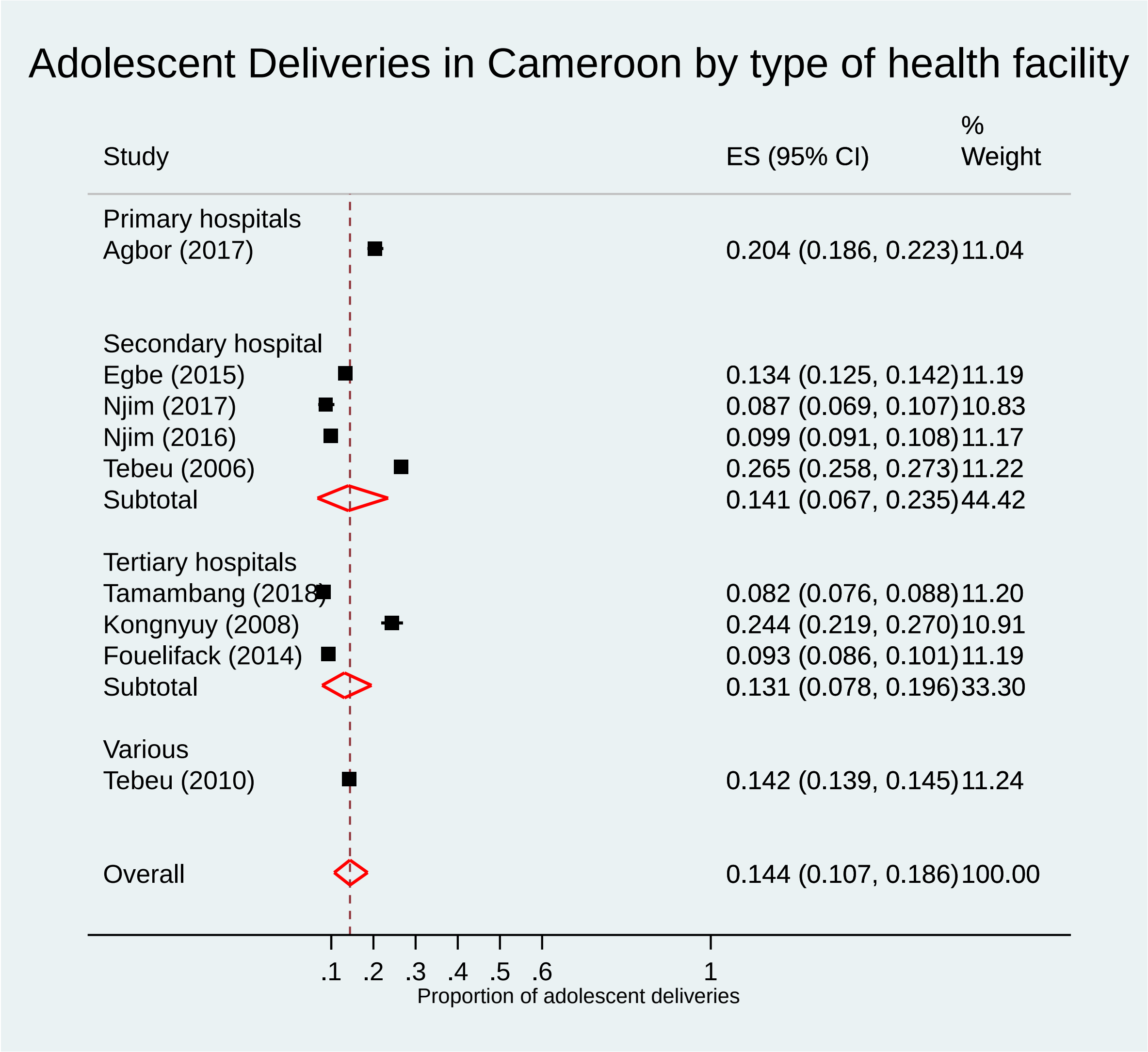

Supplement: Supplementary file 5 — Additional file 5. Meta-analysis showing prevalence of adolescent deliveries in Cameroon. Meta-analysis showing the prevalence of adolescent deliveries by early vs late adolescent deliveries, setting and type of health facility. [file 13690_2020_406_MOESM5_ESM.pdf]
