# Supplementary material for: Prevalence of adolescent deliveries and its complications in Cameroon: a systematic review and meta-analysis
Source: Arch Public Health. 2020 May 5;78:24. doi: 10.1186/s13690-020-00406-1 (PMC7199297; doi:10.1186/s13690-020-00406-1)

Meta-analysis of the association between marital status and adolescent deliveries in Cameroon

Author	Adolescents marital status - single	Adolescents Total	Non-adolescents marital status - single	Non-adolescents Total	weight	Odds ratio [95% CI]
Agbor 2017	218	368	220	1435	16.9%	8.03 [6.23; 10.33]
Egbe 2015	95	148	115	360	16.7%	3.82 [2.55; 5.71]
Njim 2017	72	77	276	809	15.9%	27.81 [11.10; 69.64]
Njim 2016	318	491	1248	4450	16.9%	4.72 [3.88; 5.74]
Kongnyuy 2008	19	268	344	832	16.7%	0.11 [0.07; 0.18]
Fouelifack 2014	432	544	2654	5301	16.9%	3.85 [3.10; 4.77]
**Total (fixed effect, 95% CI)**		**1896**		**13187**	**--**	**3.16 [2.85; 3.51]**
**Total (random effects, 95% CI)**					**100.0%**	**3.40 [0.49; 23.53]**
**Prediction interval**						**[0.01; 810.95]**

Heterogeneity: $Tau^2 = 3.3206$; $Chi^2 = 260.64$, df = 5 (P < 0.01); $I^2 = 98\%$

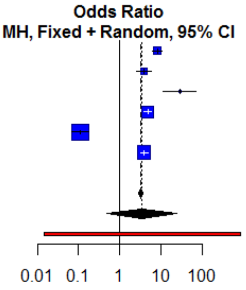

Meta-analysis of the association between gravidity and adolescent deliveries in Cameroon

Author	Adolescents primigravidas	Adolescents Total	Non-adolescents primigravidas	Non-adolescents Total	weight	Odds ratio [95% CI]
Agbor 2017	282	363	162	1400	25.0%	26.61 [19.78; 35.79]
Egbe 2015	108	148	114	360	23.5%	5.83 [3.81; 8.91]
Tamambang 2018	521	662	1545	7394	25.9%	13.99 [11.52; 16.99]
Fouelifack 2014	390	510	1085	4627	25.7%	10.61 [8.55; 13.16]
**Total (fixed effect, 95% CI)**		**1683**		**13781**	**--**	**13.04 [11.52; 14.76]**
**Total (random effects, 95% CI)**					**100.0%**	**12.45 [4.63; 33.47]**
**Prediction interval**						**[0.69; 225.82]**

Heterogeneity: $Tau^2 = 0.3571$; $Chi^2 = 39.96$, df = 3 (P < 0.01); $I^2 = 92\%$

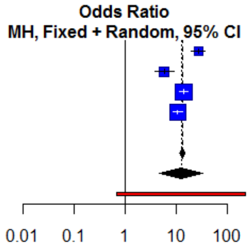

Meta-analysis of the association between parity and adolescent deliveries in Cameroon

Author	Adolescents primiparas	Total	Non-adolescents primiparas	Total	weight	Odds ratio [95% CI]
Agbor 2017	285	363	363	1398	25.2%	10.42 [7.89; 13.75]
Tamambang 2018	92	662	662	7394	25.3%	1.64 [1.30; 2.07]
Njim 2017	61	69	69	713	24.3%	71.17 [32.70; 154.89]
Fouelifack 2014	50	510	510	4622	25.2%	0.88 [0.65; 1.19]
**Total (fixed effect, 95% CI)**		**1604**		**14127**	--	**3.06 [2.70; 3.47]**
**Total (random effects, 95% CI)**					**100.0%**	**5.59 [0.24; 128.94]**
**Prediction interval**						**[0.00; 68183.80]**

Heterogeneity: $Tau^2 = 3.8095$; $Chi^2 = 227.90$, df = 3 ($P < 0.01$); $I^2 = 99\%$

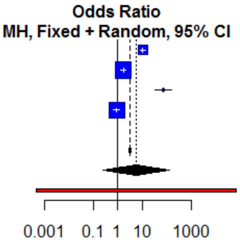

Forest plot showing the association between level of education and adolescent deliveries in Cameroon

Author	Adolescents Primary level	Total	Non-adolescents Primary level	Total	weight	Odds ratio [95% CI]
Egbe 2015	14	148	20	360	43.4%	1.78 [0.87; 3.62]
Kongnyuy 2008	99	268	96	832	56.6%	4.49 [3.24; 6.23]

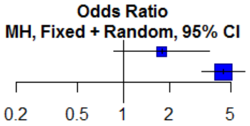

Meta-analysis of the association between employment status and adolescent deliveries in Cameroon

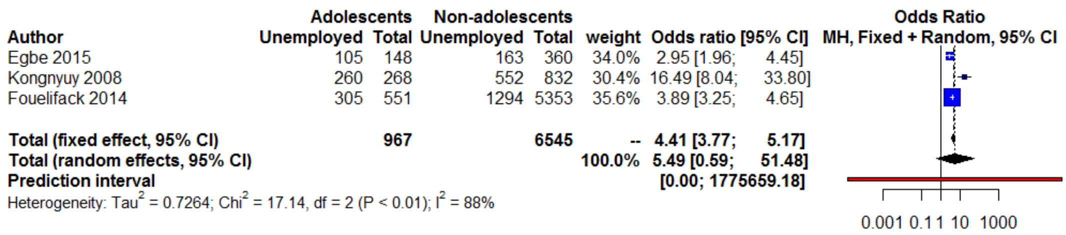

Forest plot showing the association between antenatal care visits and adolescent deliveries in Cameroon

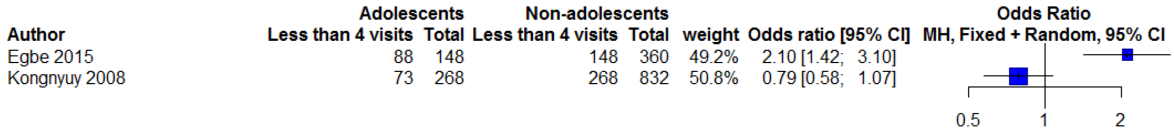

Supplement: Supplementary file 6 — Additional file 6. Meta-analysis of risk factors of adolescent deliveries. Meta-analysis of the various risk factors of adolescent deliveries in Cameroon. [file 13690_2020_406_MOESM6_ESM.pdf]
