# Supplementary material for: Prevalence of adolescent deliveries and its complications in Cameroon: a systematic review and meta-analysis
Source: Arch Public Health. 2020 May 5;78:24. doi: 10.1186/s13690-020-00406-1 (PMC7199297; doi:10.1186/s13690-020-00406-1)

Meta-analyis of association between caesarean deliveries and adolescent deliveries in Cameroon

Author	Adolescents Caesarean delivery	Adolescents Total	Non-adolescents Caesarean delivery	Non-adolescents Total	weight	Odds ratio [95% CI]
Agbor 2017	2	367	14	1435	3.0%	0.56 [0.13; 2.46]
Tamambang 2018	34	662	461	7394	20.0%	0.81 [0.57; 1.16]
Njim 2017	13	77	96	809	11.5%	1.51 [0.80; 2.84]
Njim 2016	81	491	925	4450	24.3%	0.75 [0.59; 0.97]
Kongnyuy 2008	28	268	69	832	16.3%	1.29 [0.81; 2.05]
Fouelifack 2014	92	554	784	5367	24.8%	1.16 [0.92; 1.47]
**Total (fixed effect, 95% CI)**		**2419**		**20287**	--	**0.95 [0.83; 1.10]**
**Total (random effects, 95% CI)**					**100.0%**	**1.00 [0.73; 1.37]**
**Prediction interval**						**[0.46; 2.17]**

Heterogeneity: $Tau^2 = 0.0635$; $Chi^2 = 11.05$, df = 5 (P = 0.05); $I^2 = 55\%$

Odds Ratio
MH, Fixed + Random, 95% CI

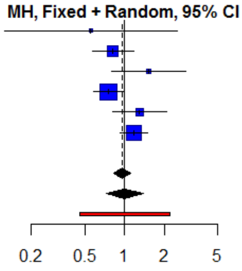

Forest plot showing association between post-partum haemorrhage and adolescent deliveries in Cameroon

Author	Adolescents Post-partum haemorrhage	Adolescents Total	Non-adolescents Post-partum haemorrhage	Non-adolescents Total	weight	Odds ratio [95% CI]
Agbor 2017	0	368	7	1803	6.7%	0.07 [0.00; 35.33]
Fouelifack 2014	5	349	69	3379	93.3%	0.70 [0.28; 1.74]

Odds Ratio
MH, Fixed + Random, 95% CI

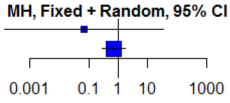

Definition of perineal tears as used in studies in meta-analysis

Author name and year	Years of patient recruitment	Type of study	Region	Age profile	Type of health facility	Setting	Definition of perineal tears
Agbor, 2017	2009 - 2016	Retrospective register analysis	North west	14 – 49	Primary hospital	Rural	Second - fourth degree
Kongnyuy, 2008	2004 - 2005	Cross-sectional study	Centre	20 – 29	Tertiary hospitals	Urban	Undefined
Fouelifack, 2014	2008 - 2010	Retrospective register analysis	Centre	27.34 ± 6.03	Tertiary hospitals	Urban	Second – fourth degree

Meta-analysis of association between perineal tears and adolescent deliveries in Cameroon

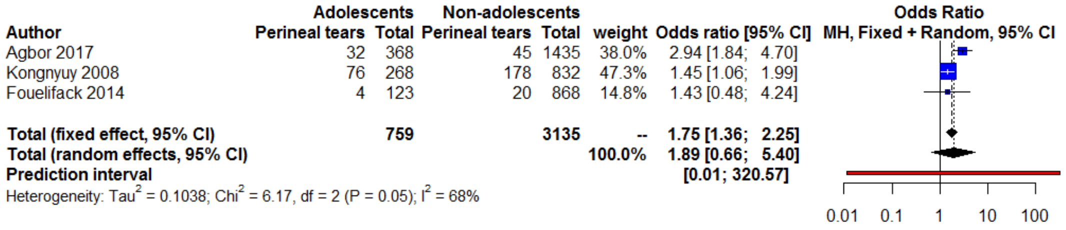

Meta-analysis of the association between operative vaginal deliveries (forceps deliveries and vacuum deliveries) and adolescent deliveries in Cameroon

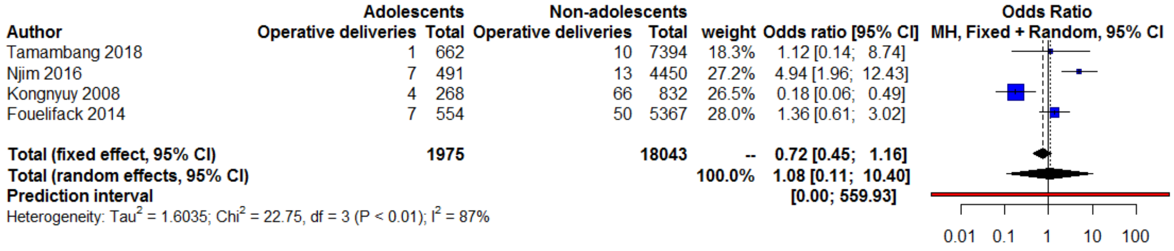

Forest plot showing association between episiotomies and adolescent deliveries in Cameroon

Author	Adolescents Episiotomy	Adolescents Total	Non-adolescents Episiotomy	Non-adolescents Total	weight	Odds ratio [95% CI]
Kongnyuy 2008	41	268	75	832	45.5%	1.82 [1.21; 2.74]
Fouelifack 2014	63	406	587	4039	54.5%	1.08 [0.81; 1.43]

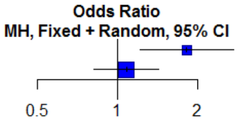

Supplement: Supplementary file 7 — Additional file 7. Meta-analysis of maternal complications of adolescent deliveries. Meta-analysis of the various maternal complications of adolescent deliveries in Cameroon. [file 13690_2020_406_MOESM7_ESM.pdf]
