# Supplementary material for: Prevalence of adolescent deliveries and its complications in Cameroon: a systematic review and meta-analysis
Source: Arch Public Health. 2020 May 5;78:24. doi: 10.1186/s13690-020-00406-1 (PMC7199297; doi:10.1186/s13690-020-00406-1)

Definition of low birth weight as used in the studies used in the meta-analysis

Author name	Sample size (N)	Years of patient recruitment	Type of study	Region	Type of health facility	Urban versus rural	Definition of low birth weight
Agbor, 2017	1803	2009 - 2016	Retrospective register analysis	North west	District health facility & Health centre	Rural	≤ 2600g
Egbe, 2015	6546	2010 - 2013	Retrospective register analysis & case-control	South west	Secondary hospital	Semiurban	< 2500g
Tamambang, 2018	8056	2010 – 2015	Retrospective register analysis	Littoral	Tertiary hospital	Urban	< 2650g
Njim, 2017	886	2015 - 2016	Retrospective register analysis	North west	Secondary hospital	Semiurban	≤ 2600g
Njim, 2016	4941	2007 - 2012	Retrospective register analysis	South west	Secondary hospital	Semiurban	≤ 2600g
Kongnyuy, 2008	1100	2004 - 2005	Cross-sectional study	Centre	Tertiary hospitals	Urban	< 2500g

Meta-analysis of the association between low birth weight and adolescent deliveries in Cameroon

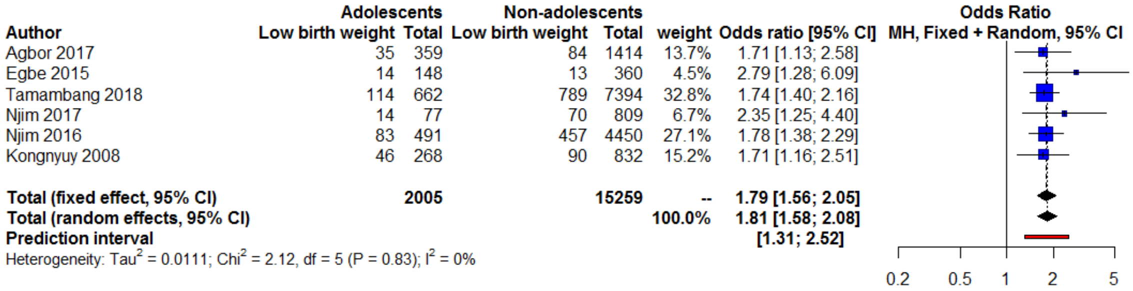

Definition of high birth weight as used in the studies included in the meta-analysis

Author name	Sample size (N)	Years of patient recruitment	Type of study	Region	Type of health facility	Setting	Definition of low birth weight
Agbor, 2017	1803	2009 - 2016	Retrospective register analysis	North west	Primary hospital	Rural	≥ 3850g
Tamambang, 2018	8056	2010 – 2015	Retrospective register analysis	Littoral	Tertiary hospital	Urban	>3850g
Njim, 2017	886	2015 - 2016	Retrospective register analysis	North west	Secondary hospital	Semiurban	≥ 3850g
Njim, 2016	4941	2007 - 2012	Retrospective register analysis	South west	Secondary hospital	Semiurban	≥ 3850g

Meta-analysis of the association between high birth weight and adolescent deliveries

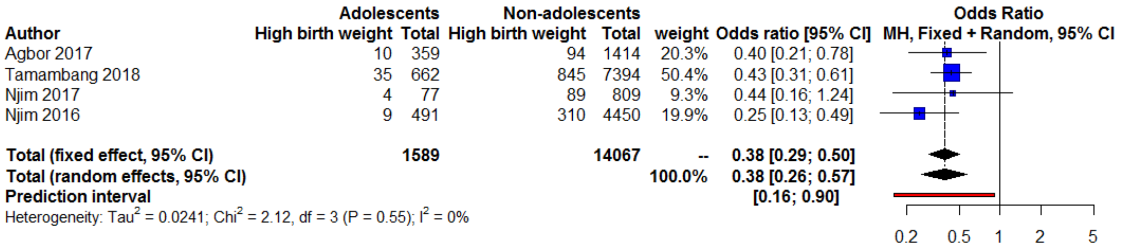

Definition of neonatal asphyxia as used in the studies in the meta-analysis

Author name	Sample size	Years of patient recruitment	Type of study	Region	Type of health facility	Setting	Definition of neonatal asphyxia
Agbor, 2017	1803	2009 - 2016	Retrospective register analysis	North west	Primary hospital	Rural	Fifth minute Apgar < 7
Egbe, 2015	6546	2010 - 2013	Retrospective register analysis & case-control	South west	Secondary hospital	Semiurban	Fifth minute Apgar < 7
Tamambang, 2018	8056	2010 - 2015	Retrospective register analysis	Littoral	Tertiary hospitals	Urban	First minute Apgar < 7
Njim, 2017	886	2015 - 2016	Retrospective register analysis	North west	Secondary hospital	Semiurban	First minute Apgar < 7
Njim, 2016	4941	2007 - 2012	Retrospective register analysis	South west	Secondary hospital	Semiurban	First minute Apgar < 7
Kongnyuy, 2008	1100	2004 - 2005	Cross-sectional study	Centre	Tertiary hospitals	Urban	Fifth minute Apgar < 7
Fouelifack, 2014	5997	2008 - 2010	Retrospective register analysis	Centre	Tertiary hospitals	Urban	Fifth minute Apgar < 7

Meta-analysis of the relationship between neonatal asphyxia and adolescent deliveries in Cameroon

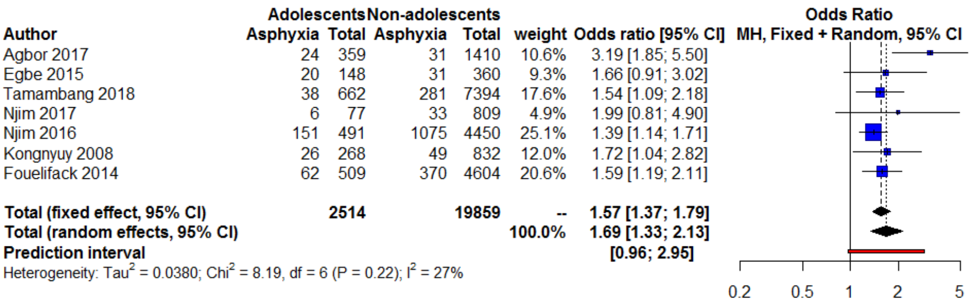

Definition of stillbirth as used in the studies in the meta-analysis

Author name	Sample size	Years of patient recruitment	Type of study	Region	Type of health facility	Setting	Definition of stillbirth
Agbor, 2017	1803	2009 - 2016	Retrospective register analysis	North west	Primary hospital	Rural	Apgar 0 at five minutes
Egbe, 2015	6546	2010 - 2013	Retrospective register analysis & case-control	South west	Secondary hospital	Semiurban	Undefined
Tamambang, 2018	8056	2010 - 2015	Retrospective register analysis	Littoral	Tertiary hospitals	Urban	Apgar 0 at first minute
Njim, 2016	4941	2007 - 2012	Retrospective register analysis	South west	Secondary hospital	Semiurban	Apgar 0 at first minute
Kongnyuy, 2008	1100	2004 - 2005	Cross-sectional study	Centre	Tertiary hospitals	Urban	Foetal death
Fouelifack, 2014	5997	2008 - 2010	Retrospective register analysis	Centre	Tertiary hospitals	Urban	Apgar 0 after five minutes

Meta-analysis of the relationship between stillbirths and adolescent deliveries in Cameroon

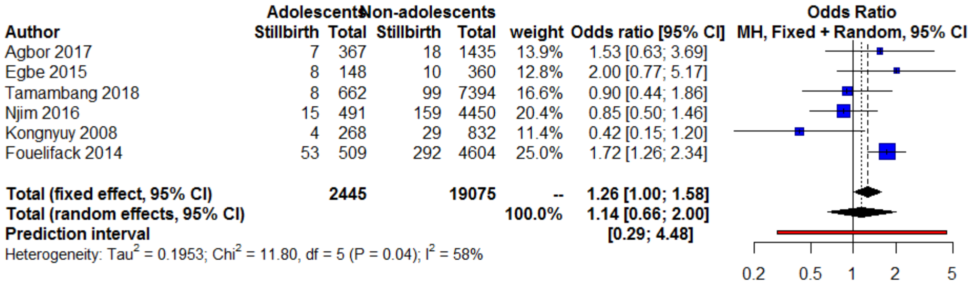

Meta-analysis of the relationship between preterm deliveries (deliveries before 37 weeks and after 28 weeks of gestation)

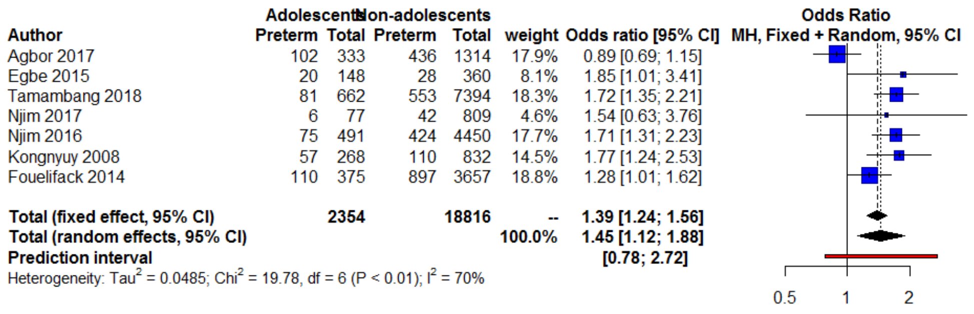

Meta-analysis of the relationship between post-term deliveries (deliveries after 42 weeks of gestation) and adolescent deliveries in Cameroon

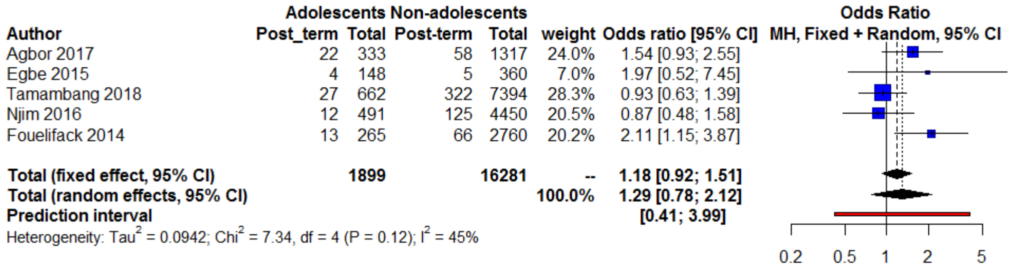

Meta-analysis of the relationship between neonatal death and adolescent deliveries in Cameroon

	Adolescents		Non-adolescents			
**Author**	**Neonatal death**	**Total**	**Neonatal death**	**Total**	**weight**	**Odds ratio [95% CI]**
Egbe 2015	6	148	9	360	26.9%	1.65 [0.58; 4.71]
Njim 2017	3	77	11	809	17.9%	2.94 [0.80; 10.78]
Kongnyuy 2008	13	268	19	832	55.2%	2.18 [1.06; 4.48]
**Total (fixed effect, 95% CI)**		**493**		**2001**	**--**	**2.10 [1.22; 3.61]**
**Total (random effects, 95% CI)**					**100.0%**	**2.13 [1.19; 3.82]**
**Prediction interval**						**[0.24; 19.03]**

Heterogeneity: $Tau^2 = 0.0113$; $Chi^2 = 0.47$, df = 2 (P = 0.79); $I^2 = 0\%$

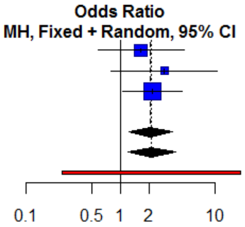

Supplement: Supplementary file 8 — Additional file 8. Meta-analysis of the foetal/neonatal complications of adolescent deliveries. Meta-analysis of the various foetal/neonatal complications of adolescent deliveries in Cameroon. [file 13690_2020_406_MOESM8_ESM.pdf]
